# Supplementary material for: Patterns of Intron Gain and Loss in Fungi
Source: PLoS Biol. 2004 Nov 30;2(12):e422. doi: 10.1371/journal.pbio.0020422 (PMC532390; doi:10.1371/journal.pbio.0020422)
Supplement: Table S1 — Also available at http://genes.mit.edu/NielsenEtAl/. (4.3 MB ZIP). [file pbio.0020422.st001.zip › NielsenEtAl/html/636.html]

AN3865.1.NCU03759.1.MG06949.1.FG05909.1


```
 CLUSTAL W (1.82) Multiple Sequence Alignments - Introns Inserted


Sequence 1: MG06949.1	859 aa
Sequence 2: FG05909.1	784 aa
Sequence 3: AN3865.1	528 aa
Sequence 4: NCU03759.1	622 aa
Alignment Length: 886 aa
Number Identitical Residues: 153 aa
Alignment Score (without introns) 10206


MG06949.1 	MERLTLPRIRKLSRLPSALTRATTLWTCSSCRHQANS----------GWRRMASTEAAKK
NCU03759.1	MDVLRTASARRTFKWQAAGILARTSWVCRSCRSQFVAPKAARRTLATSTTTTTPTAPDNK
FG05909.1 	------------------------------------------------------------
AN3865.1  	MASLRGGS--RALSWANQ-LLSRQ-RLSSKCASQRVAPPATAYSLRQYATTAKEAS---K
          	                     :     . ..  .  :. ::  :          :    .

MG06949.1 	PFYVTTPIFYVNAS~PHIGHLYTLVLADVFKRWRVLQG-EKALLCTGTDEHGMK~IQRAA
NCU03759.1	PFYVTTPIFYVNAS1PHVGHMYSMVLGDVLKRWQTLKG-NQAILCTGTDEHGTK~VQRAA
FG05909.1 	--------------~--------MVLTDVLKRWKTING-KEAYLCTGTDEHGMK~IQQAA
AN3865.1  	PYYVTTPIFYVNAA1PHVGHLYTMVLADILKRWRTLTGDNEAQLLTGTDEHGMK0GLLGE
          	.   ::.    .:: .  .   ::** *::***:.: *.::* * ******* *    . 

MG06949.1 	AASNMTPKQLCDANSEVFLDLAEKAGIDNDFFIRTTDPDHKEAVQYFWDRLWDRG----Y
NCU03759.1	IANDMDPKQFCDLNSAKFQELAAACRIDYDRFMRTTDQDHVEAVKHFWLLLKEKG----L
FG05909.1 	LKEGISPKELCDNNSNKFRDLAEVSNISHDFFIRTTDQEHKDVVQQFWLLLKARAPEGLG
AN3865.1  	IVTYAEQE-----------------------------------------MLQHRG----Y
          	       :                                          *  :.     

MG06949.1 	IYEATHKGWYCVSDETFYPESTIEKRKDPMTGEVFL~ASKETGNAVEWMEEKTYHFRLTA
NCU03759.1	IYEAKHEGWYCVSDECFYPESQLEKRQDPFTGEVYV~ASIESGNKVEWIEEKNYHFRMTA
FG05909.1 	LYKGKHEGWYCVSDECFYPEDLVEPSVVPQTGRKIM~ASTETGNEVEWIAEHTWFFPLSR
AN3865.1  	IYTSKHEGWYSVSDETFYPQSQVNLSLDPATGRKRM0VSTETGKEVEWSSETNYHFRLSA
          	:* ..*:***.**** ***:. ::    * **.  : .* *:*: ***  * .:.* :: 

MG06949.1 	LKDRLLKFFEENPEWIVPAPRMKEVVDWVENSLEDLSVSRPTSRLDWGIRVPNDESQTIY
NCU03759.1	LKDQLLEFYKNNPDWIVPQTRMNQVVDWVTNNLEDLSISRPVSRLSWGIRVPDDESQTIY
FG05909.1 	YKDKLLRFYDENPDWIQPAHRMNEVRNWVENHLEDLSVTRPTSRLNWGIRDPDDPTSTIY
AN3865.1  	FQDRLLELYKT--DFITPTHYTEQVVKSVASGLSDLSISRPVERLTWGIPVPNDNTQTIY
          	 :*:**.::.   ::* *    ::* . * . *.***::**..** ***  *:* :.***

MG06949.1 	VWIDALVNYVTKAGY-PYWTPETVQKDGWPADIHIIGKDIVR~FHCVYWPALLMALDLPL
NCU03759.1	VWVDALINYITMAGYPYWPPGREHLGG-WPVDVHVIGKDILR~FHCIYWPALLLALDLPL
FG05909.1 	VWVDALVNYLTKAGFGTKWHADDADTGIWPADVHVVGKDIIR~FHAVYWPALLMAAGLPL
AN3865.1  	VWLDALVNYLTKAGYP-FPPGEGSRLG-WPADVHVVGKDIVR2FHCVYWPAFLMALDLPL
          	**:***:**:* **:           . **.*:*::****:* **.:****:*:* .***

MG06949.1 	PRRILTHSHWTLGGKKMSKSLGNVVNPFLAMDRFGVEVIRYFLISNGRLLQDASYDNKIL
NCU03759.1	PKRILSHAHWTMEKKKMSKSIGNVVNPFYAMERFGIDTMRFYMIHNGGIANDADYSNDWL
FG05909.1 	PKKILCHNHWTMSNRKMSKSLGNVVNPFSAVQKWDLDPLRYFLMRNGSLKGDMSYSNESI
AN3865.1  	PRNVLVHAHWTMNREKMSKSTGNVVNPFFAIDRFGMDTMRFFLAHRGGLASDSDYDNRYI
          	*:.:* * ***:  .***** ******* *::::.:: :*:::  .* :  * .*.*  :

MG06949.1 	VDIYKRQLQGGVGNLLGRVMRSK--KWNVREIVERCSK------EGRPTNSNVEPKYSSF
NCU03759.1	TVEYKKHLQNGVGNLSARVTRPK--QWSLRRAVQSYQDG---NMLLQSTGEQINDSCVEH
FG05909.1 	MVIYEKELQANIGNLFNRISRNKNFSWSTLGAVQAARNGDFDNIESSVPRGDAAPDFFSL
AN3865.1  	IRDYKKYLQQGLGNIAHRTIGCA--KGKLRDYIINATSG---NSPATADDQAYQK-----
          	   *:: ** .:**:  *       . .    :    ..   .                 

MG06949.1 	EQRLFQLPGTVVDAFGELDPGAALRSIADMATE0TNKFITDCAPWSVTNENDADAAELNV
NCU03759.1	ITRLDKLASTIDNEMLQLNPSHALQKIMGLIGE0TNAFISNAEPWVIVKKQDSEALVD--
FG05909.1 	ESTLHDAPTTIRRDMDEINLSNALRGVFELLRE0ANRFISDTEPWKMTKSEDPNAKVL-I
AN3865.1  	--TLEQLSHKVAKCMEALDPRAALEEIVEVILL0TNKYFHNSEPWQNPEEKQ--------
          	   * . . .:   :  ::   **. :  :    :* :: :  **   :.::        

MG06949.1 	RSCIYLCAESLRIMGILLQPFVPNKSSELLDMLGVKETHRDLSHARLGADSTYGVPQVAL
NCU03759.1	-RIILTAGESLRIAGILLQPFMPDKAAQLLDLLGVENHNRTFHHARPLVDATYGTAFRTF
FG05909.1 	NWVIYECAEAVRIAGILLQPIMPTKAEELLDNLGVDRERRTVEFARKGADTNYGTPPLMT
AN3865.1  	-RVIFNAAESMRIMGILLQPFMPNKAKKLLDILKVDPSKRDLSDAGYARDSTYGEDVK--
          	   *  ..*::** ******::* *: :*** * *.  .* .  *    *:.**      

MG06949.1 	G---SSGVHSGLFPALVVEG2ISTPVYTPTQK0VRFEIDACFILVHLTGFWHLMLLTPPS
NCU03759.1	G------KEGIFPPPILDDI~KGMGPDAGSKK~HSSGNKPSSGNKKPTA-----------
FG05909.1 	NNIKPNGFQSLFPPVAAVDL~PDAPIDAPKGK~GKVKNRLGQVASSLA----------QE
AN3865.1  	-------KKILFPPLALEN-~-----------~---------------------------
          	        .  : *    :                                         

MG06949.1 	FLPA~ISTDQILLVPYDKHHVVTYHRWMEDP0AIQEATASEPLTLEEEYENQQSWRDSHD
NCU03759.1	----~--------------------------~----------------------------
FG05909.1 	ARET1ISTSKALLVPYEAHHVRQYHAWMQDP0DIQEATASEPMTLEEEYENQQSWRTSSD
AN3865.1  	----~--------------------------~----------------------------
          	                                                            

MG06949.1 	KLTFILCQPAKAEENKMEAGQCDSPDRMIGDINFFLYPWDEGEEDDQQTTNPEKAEYVGE
NCU03759.1	------------------------------------------------------------
FG05909.1 	KLTFIVCAPVTQDTSLVKSGTADADALMRGDINFFLYPFESDDEDD---TTDTQGWVTGE
AN3865.1  	------------------------------------------------------------
          	                                                            

MG06949.1 	IDVMVADTSQRGKGIGKAAVDAFMHYICCNQKPILQEHHGVTGEPSLRKFMVKIKADNRA
NCU03759.1	------------------------------------------------------------
FG05909.1 	VDVMIASPSHRGQGLGQAAVCALLAYIRKHIDGILAEY----GAKELKGLMVKIKEGNKG
AN3865.1  	------------------------------------------------------------
          	                                                            

MG06949.1 	SIALFRGVGFQQDGDVDYFGEVKMVLDDFGGVASKAVPGFRELTYCRTLALGGST
NCU03759.1	-------------------------------------------------------
FG05909.1 	SRALFEKLGFVQKGEVNYFGEVLMTIEWDEVSKRDWWTGAEEEFREVKYELENE-
AN3865.1  	-------------------------------------------------------
          	
```
